# Supplementary material for: Muscle Loss after Chemoradiotherapy as a Biomarker of Distant Failures in Locally Advanced Cervical Cancer
Source: Cancers (Basel). 2020 Mar 5;12(3):595. doi: 10.3390/cancers12030595 (PMC7139727; doi:10.3390/cancers12030595)
Supplement: Supplementary file 1 [file cancers-12-00595-s001.pdf]

Article

Muscle Loss after Chemoradiotherapy as a Biomarker of Distant Failures in Locally Advanced Cervical Cancer

Jie Lee, Jhen-Bin Lin, Meng-Hao Wu, Chih-Long Chang, Ya-Ting Jan and Yu-Jen Chen

Supplementary Materials

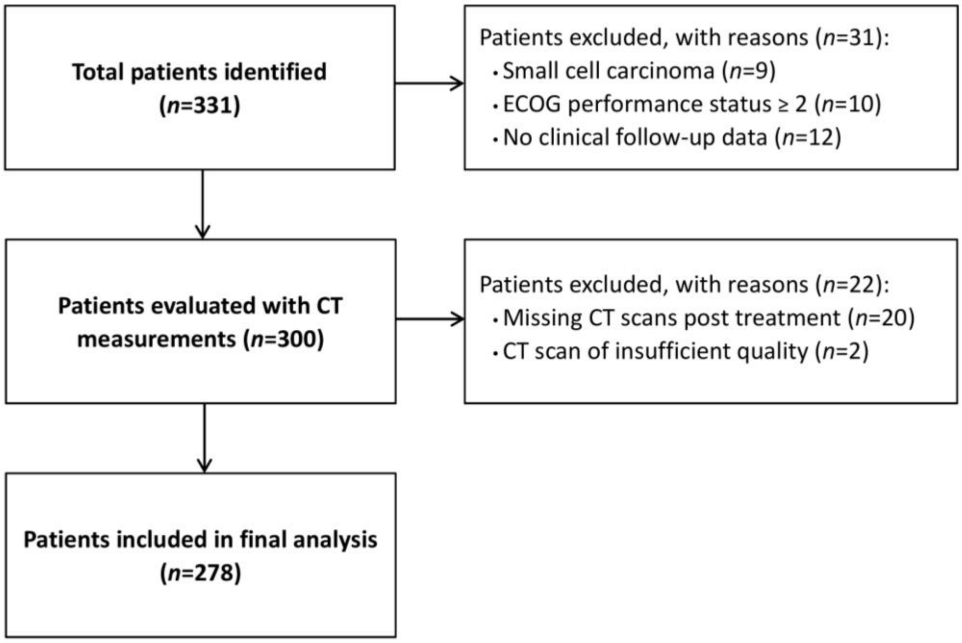

Supplementary Figure S1. Flow chart for patient inclusion.

Supplementary Table S1. Body composition parameters according to SMI change groups, values expressed as mean ± standard deviation, unless stated otherwise.

Characteristics	Overall (n = 278)	SMI loss (n = 90)	SMI maintained (n = 188)	p-value
**Pre-treatment BMI (kg/m²)**	23.4 ± 4.3	23.4 ± 4.8	23.4 ± 4.1	0.90
**Weight loss ≥ 5%, *n* (%)**	58 (20.9)	24 (26.7)	34 (18.1)	0.10
**Pre-treatment SMI (cm²/m²)**	39.8 ± 7.3	40.6 ± 7.0	39.4 ± 7.4	0.20
Pre-treatment sarcopenia[a], *n* (%)	92 (33.1)	26 (28.9)	66 (35.1)	0.30
**Post-treatment SMI (cm²/m²)**	39.3 ± 7.6	36.0 ± 6.5	40.9 ± 7.6	<0.001
Post-treatment sarcopenia[a], *n* (%)	97 (34.9)	44 (48.9)	53 (28.2)	0.001
**Pre-treatment SMD (HU)**	35.6 ± 9.5	35.6 ± 9.2	35.6 ± 9.6	0.84
Pre-treatment myosteatosis[a], *n* (%)	92 (33.1)	31 (34.4)	61 (32.4)	0.74
**SMD change (%/150 days)**	-2.9 ± 12.4	-11.2 ± 10.0	1.0 ± 11.5	<0.001
SMD maintained	154 (55.4)	23 (25.6)	131 (69.7)	<0.001
SMD loss ≥ 5%	124 (44.6)	67 (74.4)	57 (30.3)	
**Pre-treatment TATI (cm²/m²)**	97.7 ± 44.5	96.9 ± 47.8	98.0 ± 42.9	0.62
Pre-treatment low TATI[a], *n* (%)	186 (66.9)	57 (63.3)	129 (68.6)	0.38
**TATI change (%/150 days)**	-3.0 ± 15.7	-5.8 ± 16.5	-1.7 ± 15.2	0.04
TATI maintained	153 (55.0)	41 (45.6)	112 (59.6)	0.03
TATI loss ≥ 5%	125 (45.0)	49 (54.4)	76 (40.4)	

Abbreviations: BMI, body mass index; HU, Hounsfield unit; IQR, interquartile range; SMD, skeletal muscle density; SMI, skeletal muscle index; TATI, total adipose tissue index. [a] SMI < 36.3 cm²/m²,

SMD < 30.7 HU, and TATI < 112.2 cm²/m² were defined as sarcopenia, myosteatosis, and low TATI, respectively.

Supplementary Table S2. Change of body composition parameters during treatment.

	First CT scan	Second CT scan	Relative Change per 150 days (%)		
**Variable**	**Mean ± SD**	**Mean ± SD**	**Mean**	**95% CI**	***p*-value**
**BMI (kg/m²)**					
SCC	23.3 ± 4.4	22.9 ± 4.3	−1.9	−2.5 to −1.4	<0.001
Adenocarcinoma	24.1 ± 3.9	23.6 ± 3.7	−2.0	−3.6 to -0.3	0.03
**SMI (cm²/m²)**					
SCC	39.9 ± 7.3	39.6 ± 7.5	−0.5	−1.6 to 0.7	0.45
Adenocarcinoma	39.0 ± 7.2	37.3 ± 8.1	−5.0	−9.1 to −0.9	0.02
**SMD (HU)**					
SCC	35.6 ± 9.5	34.6 ± 9.6	−2.4	−4.0 to −0.9	0.002
Adenocarcinoma	35.8 ± 9.2	33.5 ± 9.4	−6.7	−11.3 to −2.2	0.005
**TATI (cm²/m²)**					
SCC	97.7 ± 45.3	93.7 ± 42.8	−3.2	−5.2 to −1.1	0.002
Adenocarcinoma	97.1 ± 38.3	93.7 ± 33.8	−2.1	−7.0 to 2.7	0.38

SCC, squamous cell carcinoma.

Supplementary Table S3. Patterns of failure according to body composition groups.

	Distant failure (total)	*p*-value	Distant failure alone	*p*-value	Distant plus Pelvic failure	*p*-value	Pelvic failure alone	*p*-value
**Weight change**		0.63		0.81		0.70		0.35
Weight maintained (*n* = 220)	43 (19.5)		35 (15.9)		8 (3.6)		7 (3.2)	
Weight loss ≥ 5% (*n* = 58)	13 (22.4)		10 (17.2)		3 (5.2)		0 (0.0)	
**SMI change**		<0.001		<0.001		<0.001		0.22
SMI maintained (*n* = 188)	18 (9.6)		17 (9.0)		1 (0.5)		3 (1.6)	
SMI loss ≥ 5% (*n* = 90)	38 (42.2)		28 (31.1)		10 (11.1)		4 (4.4)	
**SMD change**		<0.001		0.02		0.003		0.25
SMD maintained (*n* = 154)	19 (12.3)		18 (11.7)		1 (0.6)		2 (1.3)	
SMD loss ≥ 5% (*n* = 124)	37 (29.8)		27 (21.8)		10 (8.1)		5 (4.0)	
**TATI change**		0.08		0.12		0.55		0.13
TATI maintained (*n* = 153)	25 (16.3)		20 (13.1)		5 (3.3)		6 (3.9)	
TATI loss ≥ 5% (*n* = 125)	31 (24.8)		25 (20.0)		6 (4.8)		1 (0.8)	

Abbreviations: SMD, skeletal muscle density; SMI, skeletal muscle index; TATI, total adipose tissue index. Data are expressed as the absolute number of events (%).

Supplementary Table S4. Univariable Cox proportional hazards model for 3-year distant recurrence-free survival (n = 278).

	Distant recurrence-free survival	
**Variable**	**Hazard ratio (95% CI)**	***p*-value**
**Age, continuous**	1.00 (0.98–1.02)	0.92
**ECOG performance status**		
0	Reference	
1	0.85 (0.30–2.35)	0.75
**Pre-treatment BMI, continuous**	0.99 (0.93–1.06)	0.79
**BMI change, per 1%/150 days decrease**	1.03 (0.97–1.11)	0.31
**Weight loss (≥ -5.0%)**	1.17 (0.60–2.28)	0.65
**Pre-treatment SMI, categorical**		
Non-sarcopenia	Reference	
Sarcopenia[a]	1.05 (0.58–1.89)	0.87
**SMI change, per 1%/150 days decrease**	1.11 (1.08–1.14)	<0.001
**SMI change, categorical**		
SMI maintain	Reference	
SMI loss (≥ –5.0%)	7.76 (4.11–14.67)	<0.001
**Pre-treatment SMD, categorical**		
Non-myosteatosis	Reference	
Myosteatosis[a]	0.94 (0.52–1.71)	0.85
**SMD change, per 1%/150 days decrease**	1.04 (1.01–1.06)	0.002
SMD maintain	Reference	
SMD loss (≥ –5.0%)	2.39 (1.34–4.28)	0.003
**Pre-treatment TATI[a], categorical**		
High TATI	Reference	
Low TATI[a]	0.70 (0.39–1.23)	0.21
**TATI change, per 1%/150 days decrease**	1.02 (1.01–1.03)	0.04
TATI maintain	Reference	
TATI loss (≥ –5.0%)	1.64 (0.93–2.88)	0.09
**FIGO stage**		
IB-II	Reference	
III-IVA	3.89 (2.21–6.83)	<0.001
**PLNs involvement**		
Negative	Reference	
Positive	2.92 (1.57–5.42)	0.001
**Radiation field**		
Pelvic radiotherapy	Reference	
Extended-field radiotherapy	0.76 (0.43–1.34)	0.35
**Pathology**		
Squamous cell carcinoma	Reference	
Adenocarcinoma	3.83 (2.06–7.13)	<0.001
**SCC-Ag level, continuous**	1.02 (1.01–1.02)	<0.001
**Chemotherapy**		
No	Reference	
Yes	1.02 (0.43–2.40)	0.96

Abbreviations: BMI, body mass index; CI, confidence interval; ECOG, Eastern Cooperative Oncology Group; FIGO, International Federation of Gynaecology and Obstetrics; HU, Hounsfield unit; PLN, pelvic lymph node; SCC-Ag, squamous cell carcinoma antigen; SMD, skeletal muscle density; SMI, skeletal muscle index; TATI, visceral adipose tissue index. [a] SMI < 36.3 cm^2/m^2, SMD < 30.7 HU, and TATI < 112.2 cm^2/m^2 were defined as sarcopenia, myosteatosis, and low TATI, respectively.

Supplementary Table S5. Univariable and multivariable Cox proportional hazards model for 3-year distant recurrence-free survival for patients with squamous cell carcinoma (n = 246).

	Univariable		Multivariable	
**Variable**	**Hazard ratio (95% CI)**	***p*-value**	**Hazard ratio (95% CI)**	***p*-value**
**Age, continuous**	1.01 (0.98–1.03)	0.55		
**ECOG performance status**				
0	Reference			
1	1.15 (0.41–3.26)	0.79		
**Pre-treatment BMI, continuous**	0.97 (0.90–1.06)	0.50		
**Weight loss (≥ -5.0%)**	1.38 (0.65–2.94)	0.41		
**Pre-treatment SMI, categorical**				
Non-sarcopenia	Reference			
Sarcopenia	1.06 (0.53–2.14)	0.86		
**SMI change**				
SMI maintain	Reference		Reference	
SMI loss (≥ –5.0%)	6.71 (3.28–13.74)	<0.001	5.86 (2.85–12.08)	<0.001
**Pre-treatment SMD, categorical**				
Non-myosteatosis	Reference			
Myosteatosis	0.76 (0.37–1.59)	0.47		
**SMD change**				
SMD maintain	Reference			
SMD loss (≥ –5.0%)	1.69 (0.87–3.28)	0.12		
**Pre-treatment TATI[a], categorical**				
High TATI	Reference			
Low TATI	0.63 (0.33–1.24)	0.18		
**TATI change**				
TATI maintain	Reference		Reference	
TATI loss (≥ –5.0%)	2.26 (1.14–4.48)	0.02	2.26 (1.11–4.62)	0.03
**FIGO stage**				
IB-II	Reference		Reference	
III-IVA	5.00 (2.54–9.84)	<0.001	2.76 (1.35–5.67)	0.01
**PLNs involvement**				
Negative	Reference		Reference	
Positive	3.35 (1.57–7.15)	0.002	2.44 (1.09–5.46)	0.03
**Radiation field**				
Pelvic radiotherapy	Reference			
Extended-field radiotherapy	0.91 (0.47–1.76)	0.77		
**SCC-Ag level, continuous**	1.02 (1.01-1.03)	<0.001	1.02 (1.01–1.03)	<0.001
**Chemotherapy**				
No	Reference			
Yes	0.91 (0.35–2.34)	0.84		

Abbreviations: BMI, body mass index; CI, confidence interval; ECOG, Eastern Cooperative Oncology Group; FIGO, International Federation of Gynaecology and Obstetrics; HU, Hounsfield unit; PLN, pelvic lymph node; SCC-Ag, squamous cell carcinoma antigen; SMD, skeletal muscle density; SMI, skeletal muscle index; TATI, total adipose tissue index. [a] SMI < 36.3 cm^2/m^2, SMD < 30.7 HU, and TATI < 112.2 cm^2/m^2 were defined as sarcopenia, myosteatosis, and low TATI, respectively.

© 2020 by the authors. Licensee MDPI, Basel, Switzerland. This article is an open access article distributed under the terms and conditions of the Creative Commons Attribution (CC BY) license (http://creativecommons.org/licenses/by/4.0/).
